# MTHFR gene polymorphisms and their biochemical associations with serum vitamin D and IL-17 in ulcerative colitis

**DOI:** 10.5937/jomb0-62831

**Published:** 2026-05-22

**Authors:** Guang Chen, Meihong Yan, Xiaoyang Hu, Hanfei Xu, Shengai Piao

**Affiliations:** 1 First Affiliated Hospital Heilongjiang University of Chinese Medicine, Hospital Office, Harbin, China; 2 Harbin Medical University Cancer Hospital, Harbin, China; 3 Basic Medical College of Heilongjiang University of Chinese Medicine, Harbin, China; 4 First Affiliated Hospital Heilongjiang University of Chinese Medicine, Department of Infection Control, Harbin, China; 5 Second Affiliated Hospital Heilongjiang University of Chinese Medicine, Department of Pediatrics, Harbin, China

**Keywords:** MTHFR, ulcerative colitis, gene polymorphism, vitamin D, IL-17, homocysteine metabolism, MTHFR, ulcerozni kolitis, genski polimorfizam, vitamin D, IL-17, metabolizam homocisteina

## Abstract

**Background:**

Disturbances in homocysteine metabolism and immune-inflammatory pathways may contribute to the pathogenesis of ulcerative colitis (UC). Methylenetetrahydrofolate reductase (MTHFR) plays a central biochemical role in homocysteine remethylation, and its genetic variants may influence downstream biomarkers such as vitamin D and interleukin-17 (IL-17). This study examined the associations of three MTHFR polymorphisms (rs110298, rs132981, rs167281) with UC susceptibility, disease characteristics, and related biochemical markers.

**Methods:**

A total of 124 UC patients and 128 healthy controls were enrolled. Genotyping of MTHFR SNPs was performed by PCR amplification followed by ligase detection reaction. Serum 25-hydroxyvitamin D was measured using electrochemiluminescence immunoassay, and IL-17 levels were quantified by ELISA. Associations between MTHFR genotypes, UC onset, disease location, severity, and biochemical markers were statistically evaluated.

**Results:**

All polymorphisms conformed to Hardy-Weinberg equilibrium. The rs110298 GG genotype and rs132981 TT genotype were significantly more frequent in UC patients than controls (P &lt; 0.05), whereas rs167281 showed no association with UC. Both rs110298 and rs132981 were linked to UC disease location and severity (P &lt; 0.05). UC patients exhibited significantly reduced serum vitamin D and elevated IL-17 levels compared with controls (P &lt; 0.05). Importantly, rs110298 (AG/GG) and rs132981 (TT) genotypes were associated with lower vitamin D and higher IL-17 concentrations in UC patients (P &lt; 0.05), indicating a biochemical effect of MTHFR variants on inflammatory and immunomodulatory pathways.

**Conclusions:**

MTHFR polymorphisms rs110298 and rs132981 are significantly associated with UC susceptibility and clinical phenotype and exert measurable biochemical effects on serum vitamin D and IL-17 levels. These findings highlight the potential value of MTHFR-related pathways as biomarkers for disease characterization and as mechanistic contributors to UC-related immune dysregulation.

## Abstract (sr)

**Uvod:**

Poremećaji u metabolizmu homocisteina i imunoinflamatornim putevima mogu doprineti patogenezi ulceroznog kolitisa (UK). Metilentetrahidrofolat reduktaza (MTHFR) igra centralnu biohemijsku ulogu u remetilaciji homocisteina, a njene genetske varijante mogu uticati na nizvodne biomarkere kao što su vitamin D i interleukin-17 (IL-17). Ova studija je ispitala povezanost tri polimorfizma MTHFR (rs110298, rs132981, rs167281) sa podložnošću UK, karakteristikama bolesti i povezanim biohemijskim markerima.

**Metode:**

Ukupno je uključeno 124 pacijenta sa ulceroznim kolitisom i 128 zdravih kontrola. Genotipizacija MTHFR SNP-ova je izvršena PCR amplifikacijom, a zatim reakcijom detekcije ligazom. Serumski 25-hidroksivitamin D je meren pomoću elektrohemiluminiscentnog imunotesta, a nivoi IL-17 su kvantifikovani ELISA metodom. Asocijacije između MTHFR genotipova, početka ulceroznog kolitisa, lokacije bolesti, težine i biohemijskih markera su statistički procenjene.

**Rezultati:**

Svi polimorfizmi su se podudarali sa Hardi-Vajnbergovom ravnotežom. Genotip rs110298 GG i genotip rs132981 TT bili su značajno češći kod pacijenata sa ulceroznim kolitisom nego kod kontrolne grupe (P &lt; 0,05), dok rs167281 nije pokazao povezanost sa ulceroznim kolitisom. I rs110298 i rs132981 bili su povezani sa lokacijom i težinom bolesti ulceroznog kolitisa (P &lt; 0,05). Pacijenti sa ulceroznim kolitisom pokazali su značajno smanjen nivo vitamina D u serumu i povišene nivoe IL-17 u poređenju sa kontrolnom grupom (P &lt; 0,05). Važno je napomenuti da su genotipovi rs110298 (AG/GG) i rs132981 (TT) bili povezani sa nižim nivoom vitamina D i višim koncentracijama IL-17 kod pacijenata sa ulceroznim kolitisom (P &lt; 0,05), što ukazuje na biohemijski efekat varijanti MTHFR na inflamatorne i imunomodulatorne puteve.

**Zaključak:**

Polimorfizmi MTHFR-a rs110298 i rs132981 su značajno povezani sa osetljivošću na ulcerozni kolitis i kliničkim fenotipom i imaju merljive biohemijske efekte na nivoe vitamina D i IL-17 u serumu. Ovi nalazi ističu potencijalnu vrednost puteva povezanih sa MTHFR-om kao biomarkera za karakterizaciju bolesti i kao mehanističkih doprinosa imunološkoj disregulaciji povezanoj sa ulceroznim kolitisom.

## Introduction

Ulcerative colitis (UC) is a chronic, nonspecific inflammatory disorder of the colonic mucosa, first described by Willks and Moxon in 1875 and later designated by Willks and Boas in 1903 [1]. Although its precise etiology remains unclear, UC is widely recognized as a multifactorial disease influenced by genetic predisposition, environmental triggers, and dysregulated immune responses [2]. For genetically susceptible individuals, alterations in intestinal microbiota can induce excessive mucosal immune activation, resulting in chronic epithelial injury and inflammation. Clinically, UC commonly affects young and middle-aged adults and is characterized by bloody mucopurulent stool, abdominal pain, diarrhea, anemia, and weight loss. UC is also associated with extra-intestinal manifestations—including dermatologic, ocular, and joint involvement—and may lead to severe complications such as toxic megacolon and colorectal cancer [3][4].

Homocysteine (Hcy) is a sulfur-containing amino acid and a key intermediate in methionine metabolism via the transsulfuration and reme-thylation pathways [5]. Although Hcy does not participate directly in protein synthesis, it plays an essential role in DNA synthesis, methylation reactions, and the production of neurotransmitters and phospholipids [6]. Methylenetetrahydrofolate reductase (MTH-FR), methionine synthase, and methionine synthase reductase are critical enzymes regulating Hcy metabolism. Among the known variants, the MTHFR C677T mutation is a major genetic determinant of mild to moderate hyperhomocysteinemia [7]. Elevated serum Hcy has been consistently reported in patients with inflammatory bowel disease (IBD) [8]; however, the relationship between MTHFR gene polymorphisms and UC susceptibility or clinical features has not yet been fully elucidated.

Vitamin D and interleukin-17 (IL-17) are also important immunomodulators implicated in UC pathogenesis. Vitamin D promotes regulatory T-cell development and IL-10 secretion, thereby suppressing Th17-mediated IL-17 production. It also directly inhibits Th17 differentiation [9]. Conversely, IL-17 is a potent pro-inflammatory cytokine that contributes to mucosal inflammation in IBD [10]. Evidence further indicates that polymorphisms in the MTHFR gene and the vitamin D receptor may interact with dietary folate and vitamin D intake, influencing disease risk in immune- and inflammation-related conditions [11]. However, whether MTHFR polymorphisms contribute to UC through altered vitamin D and IL-17 homeostasis remains unknown.

In this study, we investigated three single-nucleotide polymorphisms (SNPs) of the MTHFR gene (rs110298, rs132981, and rs167281) and evaluated their associations with UC susceptibility, disease location, and severity. We further examined whether these polymorphisms influence serum vitamin D and IL-17 levels in UC patients. Our objective was to provide biochemical and genetic evidence to better understand the molecular mechanisms underlying UC pathogenesis.

## Materials and methods

### Research subjects

A total of 114 patients with confirmed ulce-rative colitis (UC), with a mean age of 45.65 ± 3.28 years, were enrolled as the Ulcerative Colitis group. The diagnosis of UC was established based on clinical presentation, laboratory tests, radiologic assessment, endoscopic findings, and histopathological confirmation. Disease classification, lesion location, and severity grading followed the European evidence-based consensus on surgery for ulcerative colitis.

Additionally, 128 age-matched healthy individuals (mean age: 44.69 ± 1.94 years) who underwent routine physical examinations during the same period were recruited as the Control group. After an overnight fast of at least 8 hours, 4 mL of peripheral venous blood was collected from each participant, anticoagulated with ethylenediaminetetraacetic acid (EDTA), and stored at -20°C until further analysis.

### Detection of serum vitamin D

Serum 25-hydroxyvitamin D concentrations were measured using an automated electrochemilu-minescence immunoassay on the Roche E411 analyzer (Roche Diagnostics, Basel, Switzerland). All measurements were performed using commercially available assay kits with appropriate calibration curves and internal quality controls. Results were expressed in ng/mL.

### Detection of serum IL-17

Serum IL-17 levels were quantified using a double-antibody sandwich enzyme-linked immuno-sorbent assay (ELISA). Briefly, microplate wells pre-coated with human anti-IL-17 antibodies served as solid-phase capture antibodies. Samples or calibrators were added, followed by incubation with horseradish peroxidase (HRP)-conjugated detection antibodies. After washing, tetramethylbenzidine (TMB) substrate was added for color development, and the reaction was stopped with sulfuric acid. Absorbance was measured at 450 nm using a microplate reader, and cytokine concentrations were calculated from the standard curve.

### DNA extraction and polymerase chain reaction (PCR) amplification

Genomic DNA was extracted from 4 mL of EDTA-anticoagulated whole blood using a commercial extraction kit (Wuhan Servicebio Technology Co., Ltd., Wuhan, China) according to the manufacturer's instructions. DNA integrity was verified by 1.5% agarose gel electrophoresis, and concentration and purity were assessed using ultraviolet spectrophotometry.

Specific primers were designed to amplify the MTHFR polymorphisms rs110298, rs132981, and rs167281. PCR was performed in a 20 mL reaction system containing 2.0 mL DNA template, 10.0 mL 2x PCR Mix, 0.4 mL each of forward and reverse primers, and 7.2 mL nuclease-free water. The amplification protocol consisted of denaturation at 95°C for 120 s; 35 cycles of 94°C for 30 s, 57°C for 90 s, and 72°C for 60 s; followed by a final extension at 72°C for 10 min. PCR products were confirmed by agarose gel electrophoresis. Primer sequences and product lengths are shown in Table 1.

**Table 1:** Primer sequences and product sizes in this research.

Polymorphism	Primer sequence (5'-3')	Product (bp)
rs110298	Forward: ACGTAGTGCTGTAGTCGT	189
	Reverse: TGTGTCGACCCCTGATAC	
rs132981	Forward: AGCTGATGTCCATGTCGTG	144
	Reverse: TGTAGTCGTAGAAACGTAC	
rs167281	Forward: ACGTAGTCGTAGTGCTGTAG	191
	Reverse: AGTCGTAGTGCTGATGCTGT	

### Ligase detection reaction

Upstream and downstream probes for each polymorphic site were synthesized by BGI (Shenzhen, China). Upstream probes were phosphorylated at the 5' terminus and combined to form a 12.5 pmol/mL probe mixture. The ligase detection reaction was conducted in a 3.05 mL system containing 0.05 mL ligase, 1 mL reaction buffer, 1 mL PCR product, and 1 mL probe mixture. Cycling conditions were as follows: 95°C for 120 s, followed by 30 cycles of 94°C for 15 s and 50°C for 25 s.

The oligonucleotide products were quantified using ultraviolet spectrophotometry, and genotyping was performed by BGI through sequencing and fragment analysis. Data interpretation was conducted using GeneMapper software. Probe sequences and corresponding product sizes are provided in Table 2.

**Table 2:** Probe sequences of ligase reaction and product sizes of different MTHFR gene polymorphisms.

Polymorphism	Probe	Probe sequence (5'-3')	Product (bp)
rs110298	rs110298	P-TGTCGTAGTCGTAGTGCCCTTTTTTTTT-FAM	289
	rs110298-A	TAGCTGATGCTGATGTCGTCAGTCTTTTTTTAT	
	rs110298-G	TTACGTAGTCGTAGTCGTAGTCGTTTTTTTAAA	
rs132981	rs132981	P-CTAGTCGTAGTCGTGATCGTGTTTTTTT-FAM	199
	rs132981-C	TTTTACGTAGTCGTAGTCGTGATCGTCGTAGTC	
	rs132981-T	TTTTTTTTTTTCTAGTCGTGATTTTTAGCTGTAC	
rs167281	rs167281	P-CAGTGATGCTAGCTGTAGTCGTTTTTTT-FAM	258
	rs167281-C	TTTTTTTTTTTCGATCGTACGTAGTGTCGTGTAC	
	rs167281-G	TTTTCGTAGCTGTAGCTGAGCTTTTTACGTGAC	

### Statistical analysis

All statistical analyses were performed using SPSS version 22.0 (IBM Corp., Armonk, NY, USA). Continuous variables were tested for normality (Shapiro-Wilk test) and expressed as mean ± SD. Group comparisons were performed using independent-samples t-tests or one-way ANOVA with Bonferroni correction, while categorical variables were analyzed using chi-square or Fisher's exact tests. To control for multiple testing, Benjamini-Hochberg false discovery rate (FDR) correction was applied. Effect sizes (Cohen's d, h^2^, or odds ratios with 95% CIs) were reported where appropriate. Multivariable linear regression was used to assess the independent associations between MTHFR genotypes and serum vitamin D or IL-17, adjusting for major demographic and clinical variables.

A priori power analysis (medium effect size, a = 0.05, power = 0.80) confirmed that the total sample size (n = 252) was adequate. A two-tailed P < 0.05 was considered statistically significant.

## Results

### General clinical characteristics

There were no significant differences between the Ulcerative Colitis group and the Control group in age, sex distribution, smoking status, or alcohol consumption (P > 0.05) (Table 3). Among the 124 UC patients, disease location was classified as rectal type (n = 68), left hemicolon type (n = 16), and extensive type (n = 40). Disease severity included 48 mild, 46 moderate, and 30 severe cases.

**Table 3:** Comparisons of general clinical data between Ulcerative Colitis group and Control group.

Clinical feature	Ulcerative Colitis group<br>n=124	Control group<br>n=128	p
Age (years old)	45.65±3.28	44.69±1.94	0.509
Gender (male/female)	100/24	98/28	0.872
Smoking (%)	34%	36%	0.581
Drinking (%)	28%	29%	0.661
Disease location (%)			
Rectal type	54.8%	0	/
Left hemicolon type	12.9%	0	/
Extensive type	32.3%	0	/
Severity (%)			
Mild	38.7%	0	/
Moderate	37.1%	0	/
Severe	24.2%	0	/

### Hardy-Weinberg equilibrium analysis

All three MTHFR polymorphisms (rs110298, rs132981, and rs167281) conformed to Hardy-Weinberg equilibrium in both groups, with r^2^ values < 0.33, indicating no significant linkage disequilibrium (Table 4).

**Table 4:** Results of linkage disequilibrium test of MTHFR gene polymorphisms in each group.

Polymorphism	r^2^		
	rs110298	rs132981	rs167281
rs110298	-	0.001	0.205
rs132981	0.001	-	0.102
rs167281	0.205	0.102	-

### Associations between MTHFR genotypes and UC susceptibility

Genotype distributions revealed significant associations between UC and the MTHFR polymorphisms rs110298 and rs132981 (P < 0.05) (Table 5). Specifically, genotype GG of rs110298 and genotype TT of rs132981 were more prevalent in UC patients than in controls. In contrast, rs167281 showed no significant correlation with UC susceptibility (P > 0.05).

**Table 5:** Distribution of different genotypes of MTHFR gene polymorphisms in UC patients.

Group	rs110298			rs132981			rs167281		
	AA	AG	GG	CC	CT	TT	CC	CG	GG
Ulcerative Colitis<br>(n = 124)	19.4%	29.0%	51.6%	25.8%	22.6%	51.6%	34.4%	32.3%	32.3%
Control<br>(n = 128)	33.6%	32.8%	33.6%	32.0%	35.2%	32.8%	37.5%	32.0%	30.5%
c^2^	0.893			0.504			1.926		
P	<0.001			<0.001			0.582		

### Allelic associations with UC

Analysis of allele frequencies demonstrated significant correlations of the G allele (rs110298) and the T allele (rs132981) with UC (P < 0.05), whereas alleles of rs167281 did not differ significantly between groups (P > 0.05) (Table 6).

**Table 6:** Distribution of alleles of MTHFR gene polymorphisms in UC patients.

Group	rs110298		rs132981		rs167281	
	A	G	C	T	C	G
Ulcerative Colitis (n = 124)	33.9%	66.1%	37.1%	62.9%	51.6%	48.4%
Control (n = 128)	50%	50%	49.6%	50.4%	53.5%	46.5%
c^2^	1.984	0.829	0.332			
P	<0.001	<0.001	0.782			

### Associations between MTHFR polymorphisms and UC location

The distribution of disease location varied significantly across MTHFR genotypes (Table 7). For rs110298, the GG genotype was predominantly associated with rectal-type UC (P < 0.05), whereas AA and AG genotypes showed markedly lower proportions of left hemicolon disease (P < 0.05). For rs132981, rectal-type UC was most common among patients with CC and TT genotypes (P < 0.05), while the CT genotype showed no significant distributional pattern.

**Table 7:** Correlations of MTHFR gene polymorphisms with the location of UC.

MTHFR gene<br>polymorphism	Genotype	Rectal type<br>(n = 68)	Left hemicolon<br>type (n = 16)	Extensive type<br>(n=40)	c^2^	P
rs110298	AA (n = 24)	33.3% (8)	20.8% (5)	45.9 % (11)	1.847	0.016
	AG (n=36)	44.4% (16)	5.6% (2)	50% (18)	1.029	0.021
	GG (n = 64)	71.9% (46)	14.1% (9)	14.1% (9)	0.872	<0.001
rs132981	CC (n=32)	78.1% (25)	6.3% (2)	15.6% (5)	0.656	<0.001
	CT (n = 28)	35.7% (10)	28.6% (8)	35.7% (10)	0.661	0.452
	TT (n = 64)	51.6% (33)	9.4% (6)	39.0% (25)	0.563	0.022

### Associations between MTHFR polymorphisms and disease severity

Genotype-severity analyses (Table 8) demon-strated that: For rs110298, AA and AG genotypes were enriched in moderate UC (P < 0.05), while mild UC was most common among patients carrying the GG genotype (P < 0.05). For rs132981, moderate disease was most frequent in patients with genotype CC, whereas mild disease predominated in those with genotype TT (P < 0.05).

**Table 8:** Correlations of MTHFR gene polymorphisms with the severity of UC.

MTHFR gene<br>polymorphism	Genotype	Mild (n=48)	Moderate<br>(n=46)	Severe<br>(n = 30)	c^2^	P
rs110298	AA (n = 24)	16.7% (4)	62.5% (15)	20.8% (5)	0.874	0.001
	AG (n=36)	22.2% (8)	55.6% (20)	22.2% (8)	0.765	0.009
	GG (n = 64)	56.3% (36)	17.2% (11)	26.6% (17)	0.863	<0.001
rs132981	CC(n=32)	6.3% (2)	62.5% (20)	31.4% (10)	0.922	<0.001
	CT (n = 28)	35.7% (10)	39.3% (11)	25% (7)	0.331	0.141
	TT (n = 64)	56.3% 36)	23.4% (15)	20.3% (13)	0.458	<0.001

The CT genotype of rs132981 showed no significant relationship with disease severity (P > 0.05).

### Serum vitamin D and IL-17 levels

**Figure 1:**
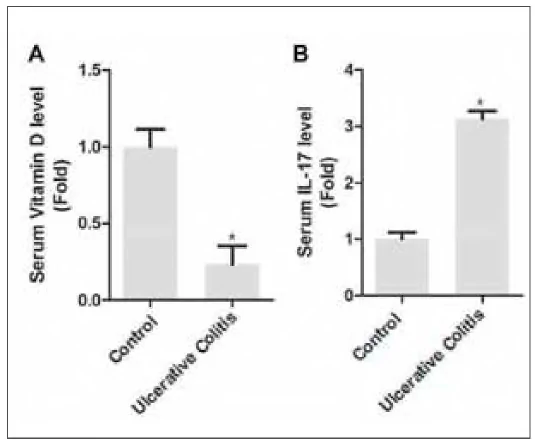
Serum vitamin D (A) and IL-17 (B) concentrations in UC patients and healthy controls.<br>Data are presented as mean ± SD. Group differences were evaluated using independent-samples t-tests. P < 0.05 after Benjamini-Hochberg correction. Units: vitamin D (ng/mL), IL-17 (pg/mL).

Compared with the Control group, UC patients had significantly lower serum vitamin D concentrations (P < 0.05) (Figure 1A) and markedly higher serum IL-17 levels (P < 0.05) (Figure 1B).

### Associations between MTHFR polymorphisms and serum biomarker levels

**Figure 2:**
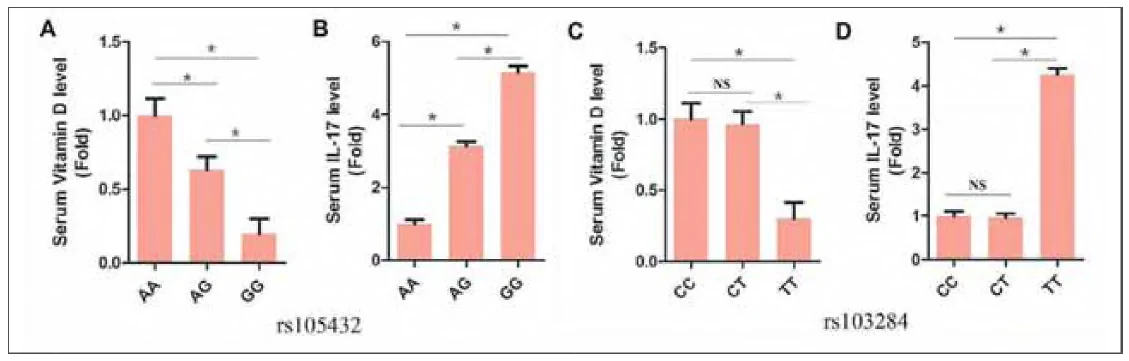
Associations between MTHFR genotypes and serum vitamin D and IL-17 levels in UC patients.<br>(A-B) rs110298; (C-D) rs132981. Data are mean ± SD. One-way ANOVA with Bonferroni post-hoc testing was used, with FDR correction for multiple comparisons. P<0.05 vs. reference genotype. NS = not significant.

Both rs110298 and rs132981 were significantly associated with alterations in serum vitamin D and IL-17 levels (Figure 2). UC patients carrying rs110298 genotypes AG and GG exhibited lower serum vitamin D and higher IL-17 levels than those with genotype AA (P < 0.05) (Figure 2A-B). For rs132981, patients with genotype TT had reduced serum vitamin D and elevated IL-17 levels compared with those carrying genotypes CC or CT (P < 0.05) (Figure 2C-D).

## Discussion

Ulcerative colitis (UC) is characterized by chronic mucosal and submucosal inflammation of the colon, yet its exact pathogenesis remains incompletely understood. Increasing evidence suggests that aberrant immune activation driven by genetic susceptibility, environmental influences, and dysbiosis contributes substantially to disease onset and progression. Among these, excessive activation of T lymphocytes has been identified as a key immunological driver of UC [12].

Although homocysteine (Hcy) concentrations were not measured in this study, the biological relevance of MTHFR polymorphisms is closely tied to their impact on folate-dependent remethylation of Hcy. Reduced MTHFR activity - particularly in variants such as rs110298 and rs132981 - can impair the generation of 5-methyltetrahydrofolate, potentially resulting in elevated Hcy levels. Hyperhomocysteinemia has been shown to enhance endothelial inflammation, promote cytokine release, and amplify Th17-mediated pathways [13][14]. Therefore, the observed associations between these SNPs, lower vitamin D levels, and increased IL-17 may reflect downstream consequences of impaired one-carbon metabolism and heightened inflammatory activation [15].

Hcy can induce inflammatory activation in monocytes, endothelial cells, and smooth muscle cells, promoting the production of chemokines (e.g., MCP-1, IL-8) and adhesion molecules (e.g., VCAM, P-selectin, E-selectin) [16]. However, Hcy levels are influenced by multiple factors, including genetic polymorphisms of Hcy-metabolizing enzymes, dietary intake of B vitamins, demographic characteristics, lifestyle habits, and comorbidities [17]. Methylenetetrahydrofolate reductase (MTHFR), a key regulatory enzyme in Hcy re-methylation, contains several functional SNPs. The common C677T polymorphism causes an alanine-to-valine substitution that reduces enzyme stability and activity, leading to elevated plasma Hcy, particularly under conditions of low folate [18][19]. The A1298C variant also reduces MTHFR activity, with in vitro studies reporting substantial decreases in enzymatic function in various genotypic combinations [20].

Consistent with these biochemical mechanisms, the present study demonstrated that the MTHFR polymorphisms rs110298 and rs132981 were significantly associated with UC susceptibility. The rs110298 GG genotype and G allele, as well as the rs132981 TT genotype and T allele, were more frequent in UC patients than in controls, suggesting a potential functional impact on Hcy metabolism. Moreover, both polymorphisms showed significant associations with UC location and disease severity, indicating that MTHFR genetic variability may influence not only disease risk but also clinical phenotype.

Vitamin D and interleukin-17 (IL-17) also play essential roles in mucosal immunity. Vitamin D, a steroid-derived hormone synthesized in the skin and metabolized in the liver and kidney, has been linked to the pathogenesis of multiple immune-mediated diseases. Epidemiological studies have shown that patients with immune disorders often exhibit lower serum vitamin D levels, and mechanistic research has demonstrated its capacity to modulate T-cell differentiation and cytokine production [21]. IL-17, a potent pro-inflammatory cytokine produced by Th17 cells, contributes to mucosal inflammation in UC and other autoimmune diseases; its activity may be regulated, at least in part, by vitamin D [22].

The findings suggest that rs110298 and rs132981 variants may contribute to biochemical and immunological heterogeneity in UC. While these polymorphisms are not proposed as diagnostic tools, they may help explain inter-individual differences in inflammatory response, vitamin D status, or disease severity. This provides a rationale for future studies evaluating whether MTHFR genotypes can inform risk stratification, nutritional optimization (e.g., folate/vitamin D supplementation), or personalized anti-inflammatory strategies.

Vitamin D status and folate metabolism are strongly influenced by external factors such as sunlight exposure, dietary intake, and supplement use. These variables were not collected in our study and may act as gene-environment modifiers of the MTH-FR-vitamin D-IL-17 axis. The absence of these data limits interpretation of the biochemical associations and should be addressed in future research.

Although mechanistically relevant, circulating homocysteine levels were unavailable and could not be correlated with genotype-biomarker relationships. This is an important limitation because Hcy is the direct metabolic substrate affected by MTHFR dysfunction. Future studies integrating genotype, Hcy levels, methylation indices, vitamin D metabolites, and cytokine profiles will be necessary to establish a more complete biochemical model.

## Conclusions

This study demonstrates, for the first time, that the MThFr gene polymorphisms rs 110298 and rs132981 are significantly associated with UC susceptibility, disease location, and clinical severity. These variants were also linked to altered levels of serum vitamin D and IL-17, suggesting that MTH-FR dysfunction may contribute to UC pathogenesis through disturbances in homocysteine metabolism and immune regulation. Taken together, these findings provide new biochemical and genetic evidence supporting the role of MTHFR-related pathways in UC. The rs110298 and rs132981 polymorphisms may represent potential biomarkers for disease risk stratification and could serve as targets for future precision-based therapeutic strategies.

## Dodatak

### Conflict of interest statement

All the authors declare that they have no conflict of interest in this work.
